# Near-fatal spontaneous hepatic rupture in a young healthy individual: a case report of successful non-surgical management and literature review

**DOI:** 10.1097/RC9.0000000000000506

**Published:** 2026-06-08

**Authors:** Jayapalanivel Vijayakumar, SatishDevakumar Murugesan, Saravanan Janakiraman, NavarathinaRajan Anburajan, Anand Lakshmanan, Jeswanth Satyanesan

**Affiliations:** Department of Surgical Gastroenterology & Liver Transplantation, Govt Stanley Medical College & Hospital, Chennai, Tamil Nadu, India

**Keywords:** atraumatic hemoperitoneum, case report, conservative management, non-surgical management, spontaneous hepatic rupture, spontaneous liver rupture, subcapsular liver hematoma

## Abstract

**Introduction and importance:**

Spontaneous hepatic rupture (SHR) is a rare but potentially fatal event, usually associated with liver tumors, pregnancy-related complications, or connective tissue disorders. Idiopathic SHR in healthy individuals is extremely rare.

**Case Presentation:**

A 19-year-old man with no history of trauma or underlying disease presented with acute epigastric pain and vomiting. The patient developed shock a few hours following admission and was shifted to the ICU. Imaging revealed a large subcapsular hepatic hematoma and hemoperitoneum. The patient was hemodynamically stabilized and managed conservatively with transfusions, antibiotics, analgesics, and close monitoring. Follow-up imaging showed gradual resolution of the hematoma. The patient remained stable during outpatient follow-up.

**Clinical discussion:**

Early diagnosis plays a crucial role, especially in cases of atraumatic hemoperitoneum. Even in the presence of shock, one should avoid rushing into surgical intervention. Initial aggressive resuscitation with blood and blood products may stabilize the patient and potentially obviate the need for major surgical procedures such as hepatectomy, as observed in this case.

**Conclusion:**

Idiopathic SHR, though rare, should be considered in atraumatic hemoperitoneum. This case is a unique example of idiopathic SHR in a young, healthy man managed entirely without surgical intervention.

## Introduction

Spontaneous rupture of the liver, first described by Abercrombie in 1844^[^1^]^, is an uncommon condition with potentially life-threatening consequences. It is most commonly associated with hepatocellular carcinoma (HCC)^[^2^]^, HELLP syndrome^[^2^]^, and connective tissue disorders such as polyarteritis nodosa and systemic lupus erythematosus^[^2^]^. However, spontaneous hepatic rupture (SHR) in healthy individuals is rarely encountered. Given the nonspecific nature of its symptoms – typically abdominal pain, vomiting, or hypotension – early diagnosis relies heavily on imaging, particularly contrast-enhanced computed tomography (CECT)^[^2^]^. While surgical intervention is often required in unstable cases, conservative treatment has shown promise in select patients^[^2^]^. Case series provide broader insights, but single case reports of rare conditions like idiopathic SHR in healthy individuals remain valuable for highlighting diagnostic and therapeutic challenges, especially when standard surgical approaches may be reconsidered.

HIGHLIGHTS

- Spontaneous hepatic rupture is a rare and life-threatening condition, especially in the absence of underlying liver pathology.
- We report a unique case of a near-fatal spontaneous liver rupture in a healthy young man, without trauma or known liver disease.
- Despite hemodynamic instability, the patient was successfully managed conservatively without surgical intervention. Timely imaging and intensive supportive care were key to favorable non-operative outcomes in this rare entity.
- This case underscores the potential for non-surgical management in select patients with spontaneous hepatic rupture.

This report highlights the successful non-operative management of a SHR in a healthy, young adult man.

## Guideline citation

This case report has been reported in line with the SCARE checklist 2025.

Kerwan A, Al-Jabir A, Mathew G, Sohrabi C, Rashid R, Franchi T, Nicola M, Agha M, Agha RA; SCARE Group. Revised Surgical Case Report (SCARE) Guideline: An update for the age of Artificial Intelligence. Premier Journal of Science. 2025;10:100 079. doi:10.70389/PJS.100079^[^3^]^.

## Case report

**Figure 1.**
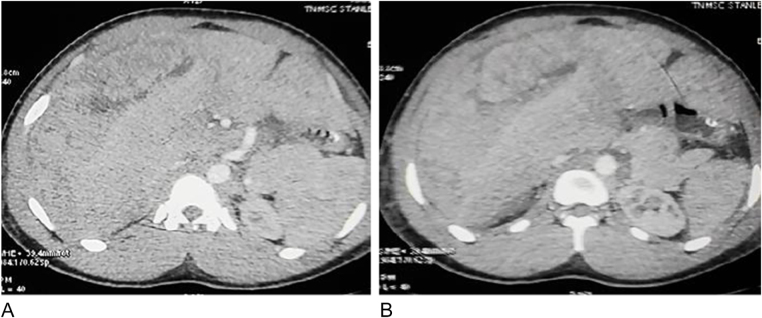
A & B: Contrast-enhanced computed tomography (CECT) of the abdomen and pelvis (early arterial phase), performed on the day of admission (07/09/2024), demonstrates a large non-enhancing subcapsular hepatic hematoma (21 × 5.2 × 18.3 cm) with associated hemoperitoneum. A heterodense lesion measuring 7.3 × 10.4 × 6 cm is noted in segment VII, with no evidence of active contrast extravasation.

A 19-year-old man presented to the Emergency Department with sudden-onset mid-epigastric pain and vomiting following household activity. There was no history of trauma, liver disease, or medication use. On examination, the patient had mid-epigastric tenderness with hepatomegaly. Initial vitals were stable. Laboratory investigations showed a hemoglobin level of 12.1 g/dl, hematocrit of 37.2%, bilirubin 0.9 mg/dl, ALT 260 U/l, and AST 220 U/l. Within 3 hours of admission, the patient developed hemorrhagic shock, manifested by hypotension (BP 80/50 mmHg), tachycardia (HR 128/min), and a drop in hemoglobin from 12.1 g/dl to 9.1 g/dl. Immediate resuscitation was initiated with intravenous crystalloids, followed by transfusion of 2 units of packed red blood cells and 2 units of fresh frozen plasma administered within the first 6 hours of admission, guided by ongoing hemoglobin monitoring and hemodynamic parameters. Hemoglobin stabilized at 10.8 g/dl post-transfusion. CECT abdomen and pelvis were performed, revealing a large non-enhancing subcapsular hepatic hematoma (21 × 5.2 × 18.3 cm) with associated hemoperitoneum. A heterodense lesion measuring 7.3 × 10.4 × 6 cm was noted in segment VII, with no evidence of active contrast extravasation (Fig. 1a and 1b). He was transferred to the ICU for close monitoring. The patient responded well to resuscitation, with stable vital signs achieved within 12 hours. He was managed conservatively with parenteral broad-spectrum antibiotics (ceftriaxone 1 g IV twice daily) and analgesics (intravenous paracetamol 1 g every 6 hours). Serial hemoglobin checks were performed every 6 hours initially, then daily, along with liver function tests. He was advised to maintain strict bed rest for the first 5 days, with gradual mobilization thereafter. Strict monitoring of abdominal girth and serial focused abdominal sonography for trauma scans were performed to ensure no further bleeding. The patient tolerated oral feeds, and his liver function tests normalized before discharge. He was discharged on day 21 and remains well on follow-up.

This case report has been reported in line with the SCARE checklist 2025.

**Figure 2.**
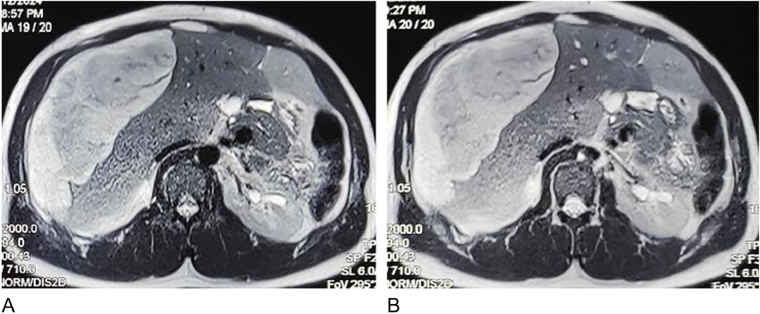
A & B: Magnetic resonance imaging (MRI) of the Abdomen and Pelvis (T2-weighted) performed one week after admission (15/09/2024), shows a well-contained subcapsular hepatic hematoma without interval expansion, consistent with stabilization under conservative management.

**Figure 3.**
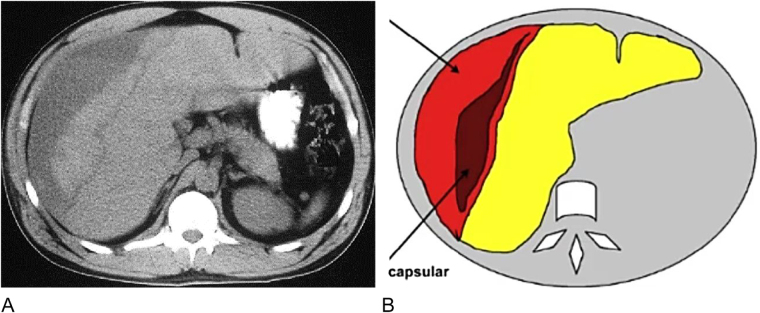
(A and B) Follow-up plain computed tomography (CT) of the abdomen and pelvis performed 1 month after presentation (9 October 2024), demonstrating a significant reduction in the size of the subcapsular hepatic hematoma and resolving hemoperitoneum.

**Figure 4.**
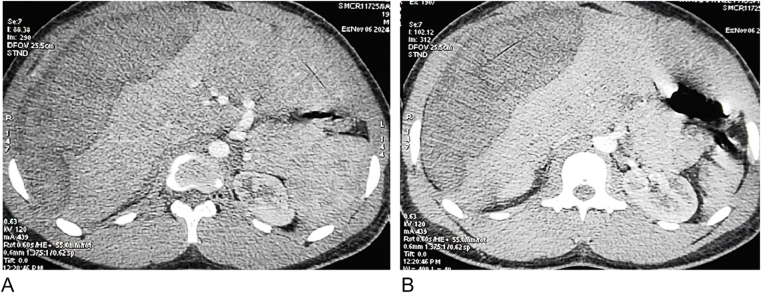
(A and B) Follow-up contrast-enhanced computed tomography (CECT) of the abdomen (early arterial phase), performed 2 months after presentation (6 November 2024), shows further interval resolution of the hepatic subcapsular hematoma with no new hemorrhage.

**Figure 5.**
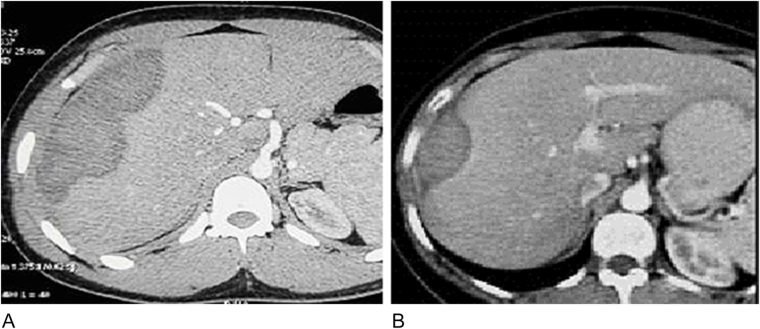
(A) Follow-up contrast-enhanced computed tomography (CECT) of the abdomen and pelvis (early arterial phase) performed 4 months after presentation (10 January 2025), demonstrating maximal resolution of the subcapsular hepatic hematoma with restoration of normal hepatic contour. (B) Follow-up contrast-enhanced computed tomography (CECT) of the abdomen and pelvis (late arterial phase) performed 5 months after presentation (6 February 2025), demonstrating near-complete resolution of the subcapsular hepatic hematoma with restoration of normal hepatic contour.

Repeat follow-up imaging (Figs. 2a,2b, 3a,3b,4a,4b,5a, and 5b) done at 1, 2, 4, and 5 months demonstrated progressive resolution of the hematoma.

## Discussion

Spontaneous hepatic hemorrhage is most commonly associated with HCC, with approximately 10% of cases presenting with rupture and bleeding^[^2^]^. Other causes include benign and malignant hepatic tumors, peliosis hepatis^[^4,5^]^, amyloidosis^[^2^]^, systemic lupus erythematosus, polyarteritis nodosa, HELLP syndrome^[^2^]^, and acute fatty liver of pregnancy. Clinical manifestations are often nonspecific and include abdominal pain, malaise, and vomiting^[^2^]^. Initial evaluation should include liver function tests, coagulation profile, and tumor markers. CECT is the diagnostic modality of choice, especially for detecting active bleeding^[^2^]^. Management aims to achieve hemostasis either conservatively or via trans arterial embolization. About 20% of patients may require surgical intervention such as perihepatic packing, hepatic artery ligation, or resection^[^2,6^]^. In stable patients with a contained hematoma and intact hepatic capsule, conservative treatment with serial imaging and monitoring can be effective^[^2^]^. In unstable cases, laparotomy with packing or resection becomes necessary. Angiographic embolization is appropriate for vascular lesions or aneurysms^[^2^]^. Liver transplantation may be considered as a last resort in cases of uncontrollable hemorrhage, although such cases are rare and unreported in the context of SHR^[^2^]^. Several pathophysiological mechanisms are implicated. In HELLP syndrome, vasospasm and endothelial injury may lead to hepatic infarction and rupture^[^2^]^. In amyloidosis, hepatic fragility and stiffness contribute to rupture risk^[^2^]^. Malignant tumors may rupture due to capsular tension from rapid tumor expansion^[^2^]^. Literature also indicates hepatotoxicity from performance-enhancing substances. Clenbuterol, a β2-agonist used illicitly for bodybuilding and weight loss, is linked to cardiovascular toxicity and liver injury^[^7^]^. Ephedra alkaloids from Ma Huang are also associated with hepatic toxicity^[^8^]^. These substances are banned in many countries^[^3,7,8^]^. Hepatotoxic herbal supplements like LipoKinetix, Kava, Chaparral, and Ma Huang have caused fulminant hepatic failure, sometimes necessitating liver transplantation^[^9,10^]^. However, this report represents a single case, which limits the generalizability of conclusions. Further case reports and multicenter case series are required to better define selection criteria and outcomes of non-operative management in SHR, particularly in patients presenting with hemodynamic instability.

## Conclusion

Spontaneous liver rupture should be considered in the differential diagnosis of atraumatic hemoperitoneum. A high index of clinical suspicion is essential for early detection and effective management. Even in the presence of shock, one should avoid rushing into surgical intervention. Initial aggressive resuscitation with blood and blood products may stabilize the patient and potentially obviate the need for major surgical procedures such as hepatectomy, as observed in this case. This case is a unique example of idiopathic SHR in a young, healthy man managed entirely without surgical intervention.

## Data Availability

Not applicable.
